# Unusual presentation of duodenal plasmablastic lymphoma in an immunocompetent patient: A case report and literature review

**DOI:** 10.3892/ol.2014.2604

**Published:** 2014-10-10

**Authors:** CHUN CAO, TING LIU, SHIFENG LOU, WEIPING LIU, KAI SHEN, BING XIANG

**Affiliations:** 1 Department of Hematology, The Second Affiliated Hospital of Chongqing Medical University, Chongqing 400010, P.R. China; 2 Department of Hematology, West China Hospital, Sichuan University, Key Laboratory of Hematology of Sichuan Province, Chengdu, Sichuan 610041, P.R. China; 3 Department of Pathology, West China Hospital, Sichuan University, Chengdu, Sichuan 610041, P.R. China

**Keywords:** plasmablastic lymphoma, immunocompetent, duodenum, Epstein-Barr virus-encoded small RNA-negative

## Abstract

Plasmablastic lymphoma (PBL) is a rare and recently described entity of large B-cell lymphoma. It predominantly occurs in the oral cavity of human immunodeficiency virus (HIV)-positive patients and exhibits a highly aggressive clinical behavior without effective treatment. Recently, sporadic cases describing PBL in extraoral locations of HIV-negative patients have been reported; frequently in patients with underlying immunosuppressive states. To develop the understanding of PBL, the current study reports the unusual presentation of duodenal PBL and reviews the pathogenesis, immunohistochemical features, clinical and differential diagnoses, as well as the treatment of PBL as described in previous studies. The case of a 75-year-old female with duodenal PBL without definite immunosuppression is presented in the current report. The tumor was composed of large B-cell-like cells, and was positive for cluster of differentiation 138 and melanoma ubiquitous mutated-1, with ~80% of the tumor cells positive for Ki-67. The features of the tumor were as follows: Extraoral location, HIV-negative, immunoglobulin M λ-type M protein expression, light chain restriction (monoclonal) and Epstein-Barr virus-encoded small RNA-negative, which are considered to be unusual for PBL. These unusual features complicate the differentiation of PBL from other plasma cell diseases. To the best of our knowledge, this is the first study to report a case of duodenal PBL in an immunocompetent patient. To date, the standard treatment of PBL remains elusive, however, the most commonly administered chemotherapy treatments are CHOP [intravenous cyclophosphamide (750 mg/m^2^, day 1), intravenous doxorubicin (50 mg/m^2^, day 1), intravenous vincristine (1.4 mg/m^2^, day 1) and prednisone (100 mg, days 1–50)]-like regimens. The patient was administered two cycles of CHOP chemotherapy for 56 days, however, ultimately succumbed as a result of disease progression. Therefore, PBL represents a diagnostic and therapeutic challenge. PBL must be considered in the differential diagnosis of gastrointestinal tumors in daily practice, even in immunocompetent patients. Furthermore, CHOP does not appear to be an optimal treatment regimen and more intensive regimens are required.

## Introduction

Plasmablastic lymphoma (PBL) has been recently recognized as an aggressive non-Hodgkin’s B-cell neoplasma, which exhibits diffuse proliferation of large neoplastic cells that resemble B immunoblasts with an immunophenotype of plasma cells (1). PBL was originally described as an extremely rare variant of diffuse large B-cell lymphoma (DLBCL). PBL occurs predominantly in human immunodeficiency virus (HIV)-seropositive individuals, with a predilection for the oral cavity (2). With improved awareness of this entity, separate case reports have described PBL in extraoral locations (3) and HIV-negative patients (4). These cases have frequently been observed in patients with an underlying immunosuppressive status, including solid organ transplant recipients, and those with lymphoproliferative or autoimmune disorders (5,6); the lack of an immunodeficiency status is rare.

PBL typically presents clinically with rapid progressive growth and is associated with poor outcomes (1). It may be poorly recognized by pathologists due to its unusual immunophenotype and the difficulty in distinguishing it from other malignant tumors. To broaden the understanding of the clinical and histopathological features of HIV-negative PBL, the current study reports a rare case of PBL that primarily involves the duodenum in an immuncompetent individual and the relevant literature was reviewed. The aim of this study was to increase understanding with regard to the clinical and histopathological features of HIV-negative PBL. Written informed consent was obtained from the patient’s family.

## Case report

A 75-year-old female was admitted to the outpatient clinic of the West China Hospital (Chengdu, China) in July 2009. The patient had a history of epigastric distension and abdominal pain that had persisted for more than one month. During physical examination, an abdominal mass was palpable in the epigastrium without lymphadenopathy or hepatosplenomegaly.

The laboratory abnormalities identified included mild anemia, an elevated lactate dehydrogenase level of 1,087 U/l (normal range, 197–401 U/l), and the detection of monoclonal immunoglobulin M (IgM)-λ-type M protein and λ-type Bence Jones protein. The differential white blood cell and platelet counts, hepatic and renal function tests, serum carcinoembryonic antigen (CEA), carbohydrate antigen (CA) 199 and CA125 levels, and immunological detection reflecting T/B-lymphocyte function were normal. In addition, serology was negative for HIV, hepatitis B virus (HBV), hepatitis C virus, EBV and the HHV8 infection. *Helicobacter pylori* (HP) was detected by light microscopy (Olympus BX51; Olympus Corporation, Tokyo, Japan) with Giemsa staining. The bone marrow was free of lymphoma and no radiological evidence of a lytic bone lesion was found.

**Figure 1:**
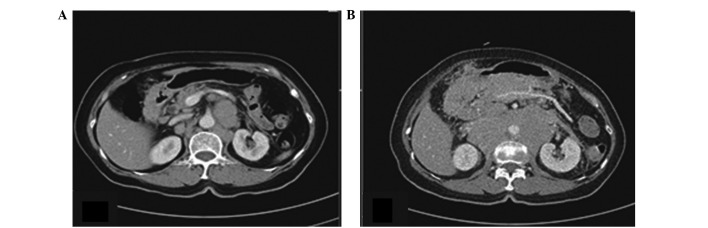
Abdominal computed tomography scan providing sequencing images at (A) diagnosis and (B) following one month of treatment. (A) MRI scan shwoing mass lesions in the wall of the gastric antrum and duodenal bulb, as well as enlarged lymph nodes in the abdominal and retroperitoneal regions. (B) Following treatment the abdominal mass enlarged with extensive lymph node metastasis.

The esophagogastroduodenoscopy revealed an ulcer in the duodenal bulb, measuring 1.2×1.5 cm in size, as well as mild chronic non-specific inflammation of the gastric antrum. An enhanced computed tomography scan of the abdomen (Fig. 1) showed mass lesions in the wall of the gastric antrum, duodenal bulb and descending segment, as well as enlarged lymph nodes in the abdominal and retroperitoneal regions (Fig. 1A). A biopsy of the ulcer mass was conducted via an endoscopy.

**Figure 2:**
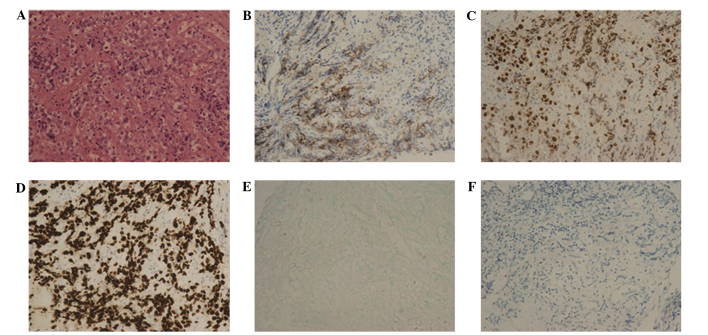
Immunohistochemical analysis of neoplastic cells (A) stained with hematoxylin and eosin, which were positive for (B) cluster of differentiation 138, (C) melanoma ubiquitous mutated-1, (D) Ki-67, (E) Epstein-Barr virus-encoded small RNA and (F) human herpesvirus 8.

The pathological results revealed the diffuse proliferation of neoplastic cells that exhibited a large B-like appearance, with a high proliferative index (Ki-67 of >80%) and frequent mitotic figures. Upon immunostaining, the neoplastic cells were positive for cluster of differentiation (CD) 138, melanoma ubiquitous mutated-1 (MUM-1), endomysial antibodies (EMA), IgM and Igλ; weakly positive for CD45 and CD10; and negative for CD19, CD20, CD3, CD5, CD79a, CD56, a B-cell-specific activator protein (PAX5), cyclin D_1_, granzyme B, B-cell lymphoma (BCL)-6, CD30, CEA, anaplastic lymphoma kinase (ALK), EBV-encoded small RNA (EBER) and HHV8 (Fig. 2). Monoclonal rearrangement of the immunoglobulin heavy chain gene and clonal restriction of the λ light chain gene expression were detected via polymerase chain reaction and heteroduplex analyses.

The PBL stage IVA was determined by the physicians from West China Hospital according to the 2008 World Health Organization classification (1). Two courses of standard-dose cyclophosphamide (750 mg/m^2^, day 1), pegylated liposomal doxorubicin (50 mg/m^2^, day 1) vincristine (1.4 mg/m^2^, day 1) and prednisone (100 mg, days 1–5) (CHOP) chemotherapy was administered, which lasted for 56 days. The abdominal mass continued to enlarge with extensive lymph node metastasis (Fig. 1B) and the patient succumbed two months following the diagnosis.

## Discussion

PBL is an uncommon type of malignancy, which most frequently arises in the oral cavity of HIV-infected patients. Subsequent to a study, which originally described PBL as an acquired immune deficiency syndrome-associated tumor in 1997 (2), a number of HIV-negative cases have been reported. These cases have frequently been found in patients with an underlying immunosuppressive status, including solid organ transplant recipients, and those exhibiting lymphoproliferative or autoimmune disorders (6). The duodenum is a rare extraoral site of involvement in PBL patients (6,7). To date, only one HIV-negative case has been reported, however, the patient had a history of HBV infection, and had previously undergone resection and radiotherapy to treat a squamous cell carcinoma of the maxillary sinus (8). To the best of our knowledge, the present study is the first to report a case of duodenal PBL in a patient without any type of immunosuppression.

PBL is significantly associated with states of immunodeficiency, particularly HIV infection (8). The EBV infection has been identified in the majority of PBL patients and observed in 74% of HIV-positive PBL cases that have been reported, worldwide (1). EBV has been demonstrated to serve an important function in the tumorigenesis of HIV-positive PBL (9). In addition, the correlation between HIV-negative PBL and EBV infection appears to be weaker than that for HIV-positive patients (5,6). Castillo *et al* (6) reported that EBER was positive in 45% of a population of HIV-negative patients. Other causes of immunodeficiency, including autoimmune disease, solid organ transplantation, steroid therapy, lymphoproliferative disease and solid tumors, may also be predisposing factors for PBL (1,6). Cases without a history of immunodeficiency have also been reported, however, these patients tended to be elderly (1). In the present case the patient underwent various assessments to exclude immunodeficiency disease and did not have a history of immunosuppression, with the exception of old age and the HP infection. Although previous studies have not established an association between the HP infection and gastrointestinal PBL, a close association between gastrointestinal mucosa-associated lymphoid tissue (MALT) lymphoma and the HP infection has been reported. These findings indicate that the HP infection and old age may cause an underlying immunosuppressive state, which renders patients susceptible to PBL. Furthermore, anti-HP treatment causes regression of low-grade B-cell gastric MALT lymphomas. However, high-grade MALT lymphoma antibiotic therapy appears to be ineffective (10). The current study failed to achieve lymphoma regression following antibiotic therapy. Further studies are required to determine whether the HP infection is a potential pathogenic factor of PBL.

Morphologically, PBL is characterized by a monomorphic proliferation of large cells resembling immunoblasts or plasmablasts. The immunophenotypic features of PBL are negativity for the typical B-cell antigens (CD20), positivity for plasma cell markers (MUM1, EMA, CD38 and CD138) and variable expression of the leukocyte common antigen (CD45) (1). All PBL characteristically exhibit a high rate of mitotic activity, as determined by the Ki-67 proliferation index, which is consistent with the findings of the current case. There is currently controversy over the cell of origin of PBL. PBL shows a close correlation with plasma cell myeloma with regard to cytogenetic variations, as well as with DLBCL in terms of genomic profiling using array comparative genomic hybridization (6). The majority of studies have found the cell of origin of PBL to be an activated, post-germinal center B-lymphocyte that has acquired a plasma cell phenotype (1, 4–7,9), however, certain studies have speculated that the cells of origin of PBL are naïve B cells (11). In the present case, MUM1 and CD138 expression was observed, however, the tumor cells were negative for B- and T-cell antigens, which was identified by the PBL immunophenotypic pattern. The lack of BCL-6 expression and M protein secretion indicated that the tumor cell may have originated from post-germinal center plasma cells. However, CD10 positivity indicated that the tumor may have stemmed from the germinal center, which is a common occurrence in PBL (1). Identification of the M protein appears to be unusual in patients with PBL, whereas monoclonal serum gammopathy has been observed in certain cases (12,13). IgM expression has been reported in a small minority of PBL cases, indicating that the M protein is not a strict discriminator (12,13). However, previous studies have proposed that PBL with light chain restriction must be reclassified as plasmablastic extramedullary plasmacytoma (EMP) (14). EMP most commonly affects the nasal cavity, paranasal sinuses and nasopharynx, and patients are typically in the fifth to seventh decade of life (15). In the present case, the tumor lesion and the highly invasive clinical progression did not support a diagnosis of EMP.

Differentiating PBL from other malignant diseases, including MM, DLBCL, poorly differentiated carcinomas, malignant melanoma, gastrointestinal stromal tumors (GIST), Burkitt’s lymphoma and anaplastic large cell lymphoma (ALCL), is diagnostically difficult. Furthermore, the treatments for these diseases are significantly different; therefore, differentiating between these entities is critical. CD20 negativity facilitates with distinguishing PBL from DLBCL and Burkitt’s lymphoma, while malignant melanoma can be excluded by observation of S-100 protein and HMB45 antigen negativity. Cytokeratin positivity aids with differentiating PBL from poorly differentiated carcinomas, and GIST is a common mesenchymal tumor of the small intestine, which is usually characterized by CD34 and CD117 expression. Furthermore, tumor cells in ALCL are consistently immunoreactive for CD30 and usually immunoreactive for CD3 and ALK. The distinction between PBL and MM is frequently dependent on their clinical presentations as PBL exhibits an identical immunophenotypic profile and an overlapping morphological spectrum with MM (multiple myeloma) (4,16). Detection of the M protein and monoclonal light chains in the blood or urine, lytic bone lesions, renal function dysfunction, hypercalcemia and anemia favors the diagnosis of a MM over PBL (1). In the present case, positivity for IgM and CD56, as well as a high Ki-67 proliferation index reduced the likelihood of a diagnosis of myeloma as an IgM myeloma is extremely rare and is characterized by CD56 negativity in the majority of cases (1). The clinical presentation of mild anemia, normal serum calcium and creatinine, as well as the lack of evidence of a lytic bone lesion did not support a diagnosis of MM in the present case.

The outcomes of PBL are usually poor, with an annual mortality rate of ~60% (7). HIV-negative patients have a poor response to chemotherapy and a reduced survival time with a median overall survival of nine versus 14 months compared with HIV-positive patients (17). Furthermore, extraoral PBL patients commonly present with disseminated disease at diagnosis (57% of patients are stage IV) (17). The optimal treatment for PBL remains elusive due to its progressive growth (6,7). Initial therapy has included lymphoma-specific multiagent systemic chemotherapy with or without consolidation radiation and hematopoietic stem cell transplantation or surgery. The most commonly adopted chemotherapeutic treatments are CHOP-like regimens, which have been administered to ~40% of chemotherapy patients, while more intensive regimens than CHOP, including cyclophosphamide, vincristine, doxorubicin and high-dose methotrexate (CODOX), ifosfamide, etoposide and high dose cytarabine (IVAC) and hyperfractionated cyclophosphamide, vincristine, doxorubicin and dexamethasone (Hyper-CVAD), have been used in 25% of chemotherapy patients (6,7). Bortezomib is a proteasome inhibitor approved for the treatment of multiple myeloma and mantle cell lymphoma; therefore, there may be a potential benefit of administering bortezomib in PBL. However, limited clinical evidence supports the use of bortezomib in PBL, with only six cases identified in a review of the literature (18,19). Of these, two patients succumbed to infectious complications and one patient succumbed to cardiopulmonary failure, while rapid disease progression was the cause of mortality in the remaining patients. In the present case, the patient refused surgery and started CHOP chemotherapy; however, the patient succumbed following two courses of CHOP chemotherapy. More intensive regimens are required for PBL, as it is a highly aggressive neoplasm and CHOP does not appear to be an optimal treatment modality.

In conclusion, PBL represents a diagnostic and therapeutic challenge. Its predominant presentation in males, a predilection for the oral cavity, history of HIV infection and EBER expression are features that may aid with its differential diagnosis. However, an extraoral location, and HIV- and EBER-negative female patients hinder the clinical differentiation. Therefore, PBL must be considered when unexplained duodenal ulcers are exhibited, even in immunocompetent individuals that do not demonstrate EBER expression.
